# Liver impalement with an antique African iron barbed spear. A case report

**DOI:** 10.1093/jscr/rjae234

**Published:** 2024-04-18

**Authors:** Barbara Y H Cervantes, Samuel E Gavor, Nuna E Jiagge, Duniesky M Lopez, Radisnay G Lambert, Fernando M Almaguer Acevedo

**Affiliations:** Department of Surgery, School of Medicine, University of Health and Allied Sciences, Ho, Sokode, P.O. Box PMB 31, Volta Region, Ghana; Department of Surgery, Ho Teaching Hospital, Ho-Denu road, Ho Municipal, P.O. Box MA 374, Volta Region, Ghana; Department of Surgery, Ho Teaching Hospital, Ho-Denu road, Ho Municipal, P.O. Box MA 374, Volta Region, Ghana; Department of Internal Medicine and Therapeutics, School of Medicine, University of Health and Allied Sciences, Ho, Sokode, P.O. Box PMB 31, Volta Region, Ghana; Department of Surgery, School of Medicine, University of Health and Allied Sciences, Ho, Sokode, P.O. Box PMB 31, Volta Region, Ghana; Department of Surgery, School of Medicine, University of Health and Allied Sciences, Ho, Sokode, P.O. Box PMB 31, Volta Region, Ghana

**Keywords:** liver impalement, penetrating abdominal injuries, hepatotomy, hepatorraphy, impalement spear

## Abstract

Impalement injuries happen when an object penetrates a body cavity or organ and remains in situ. We present a case of a 35-year-old fisherman whose act of violence resulted in the lodging of an antique iron spear in segment V of the liver, which was then referred to our institution on the day after the accident. Despite the challenges posed by patient transfer, diagnosis, resuscitation, and, most importantly, handling in the operating room, the object was successfully removed via hepatotomy, and the patient is now in good health. Impalement by an ancient African iron spear, repurposed as a fishing tool in modern times, remains undocumented in the literature, necessitating reporting and a call for further research by the medical community into managing impalement injuries of varying severity.

## Introduction

Impalement injuries happen when an object penetrates a body cavity or organ and remains in situ [1, 2]. These are among the most severe types of penetrating injuries and frequently result in significant morbidity and mortality due to vascular and visceral damage [1, 3]. Management of impaled objects is debatable because they present unique difficulties with pre-hospital treatment, transportation, and the use of suitable surgical techniques in the operating room [1]. We presented a unique case involving an antique African iron barber spear impaled in the liver and the challenges in managing it. The medical literature only reports a few cases [2, 4] none of which were caused by this specific tool.

## Case description

**Figure 1:**
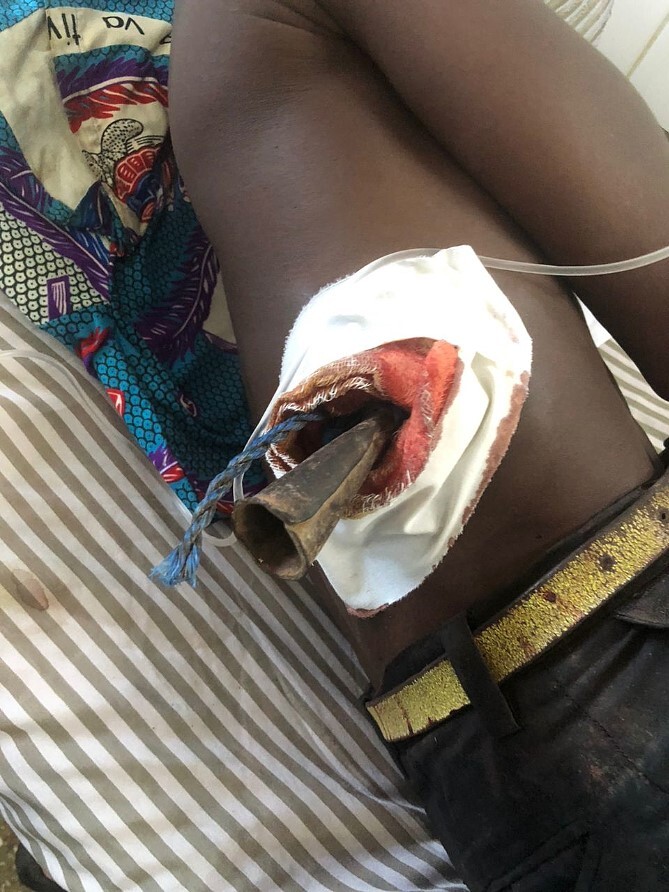
Posterolateral view of the patient with stacked object.

Our facility received a 35-year-old fisherman patient from a rural area who had undergone treatment for a penetrating abdominal trauma the day before the referral. Upon presentation, we observed the patient lying in the left lateral decubitus position with a conic metal foreign body, Insite, tied to a rope, forcing its position (Fig. 1). The object's entry point was on the right posterior wall of the abdomen, somewhere between the paravertebral muscle and the costal margin. Upon a simple inspection, we identified it as an antique African iron spear used for fishing, noting minimal external bleeding. The patient's hemodynamic were stable at the time of its presentation and so remained until the surgical act; the temperature was 37°C. Despite the patient's position, we could appreciate a non-distended abdomen with diffuse tenderness at palpation but no rebound tenderness.

**Figure 2:**
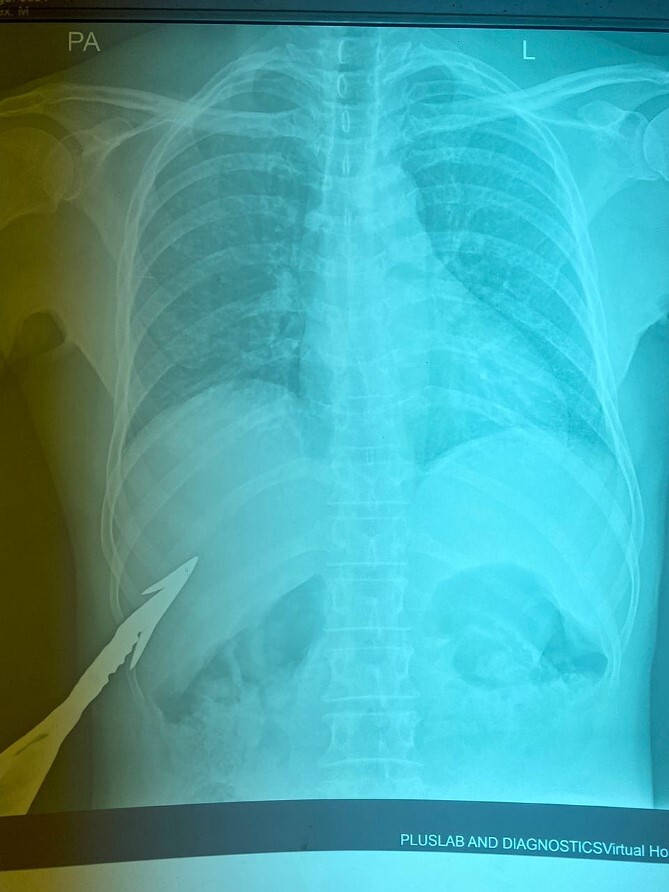
Erect PA chest X-ray (impaled spear in the right hypochondrium).

**Figure 3:**
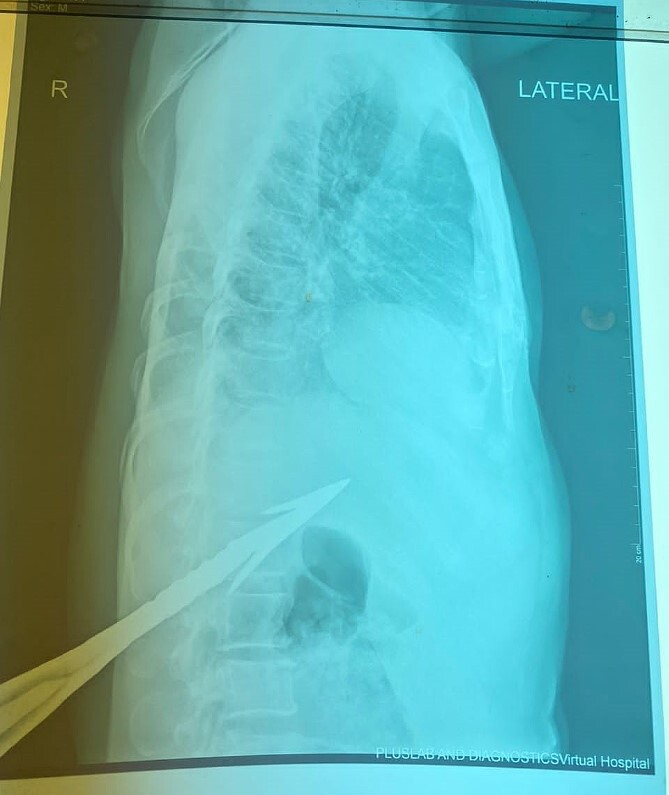
Lateral chest X-ray (impaled spear).

**Figure 4:**
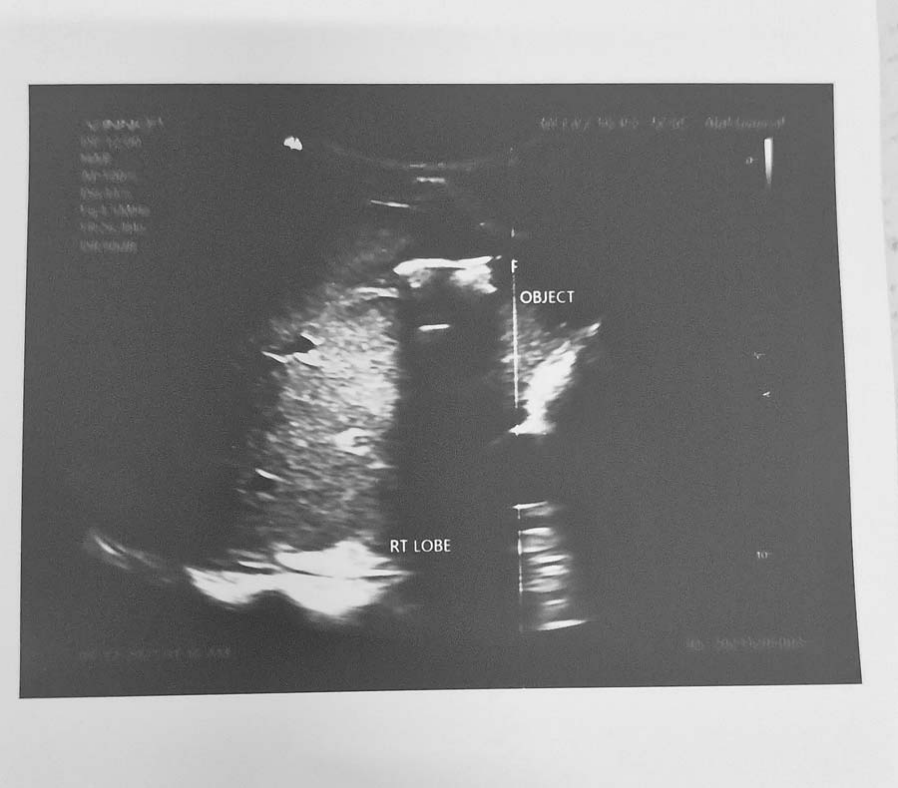
Abdominal ultrasound showing an object with an acoustic shade inside the liver parenchyma.

HB 9.9 mg/ml, sodium 135 mmol/L, chlorine 103 mmol/L, potassium 3.8 mmol/L, urea 4.3 mmol/L, and creatinine 108.5 umol/L. During the erect chest X-ray, the medical team observed the head of the spear within the abdominal cavity (Fig. 2), and a lateral chest X-ray confirmed its position in an ascending orientation (Fig. 3). The abdominal ultrasound showed the presence of a foreign body with an acoustic shade inside the liver parenchyma (Fig. 4); no free fluid was found in the Morrison’s pouch, but about 90 ml of free fluid was shown in the Douglass pouch. Both pleural spaces were free of fluid and had no pericardial fluid. We evaluated and discussed the possibility of performing an abdominopelvic computed tomography, but it was not feasible due to the patient's financial constraints.

**Figure 5:**
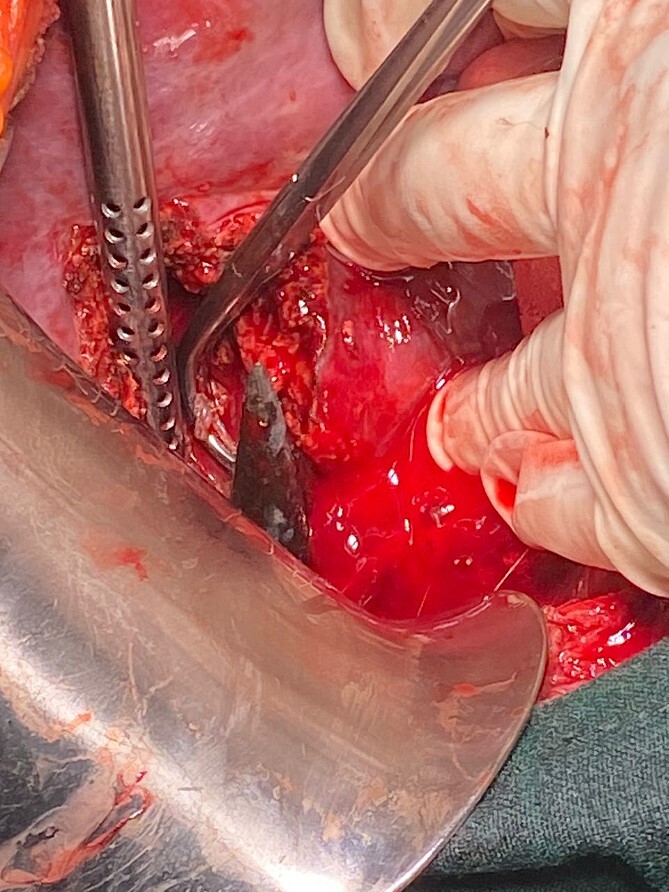
Shows head of the spear during the hepatotomy.

**Figure 6:**
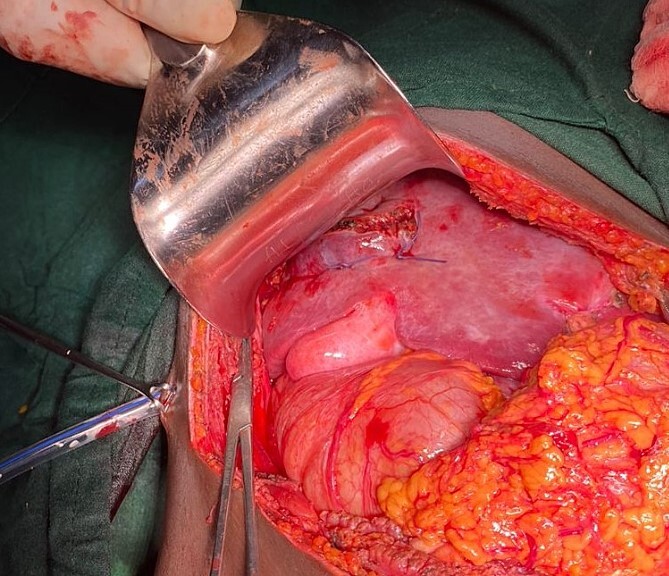
Shows hepatorraphy at segment V of the liver.

Prior to surgery, the surgical team administered a broad-spectrum antibiotic and a tetanic toxoid reactivation vaccine. An exploratory laparotomy was performed through a rooftop incision, establishing by palpation that the entire head of the spear was inside the segment V of the liver; the hepatic vascular pedicle was secured by a Pringle maneuver with Penrose drainage (in the case haemorrhage control had been necessary). We performed a hepatotomy to extract the foreign body, which was 2 cm deep from the anterior capsule and had a length of ~4 cm (Fig. 5). During the spear extraction, we ligated a biliary duct and secured haemostasis using ligature and diathermia. We performed hepatorraphy using a mattress stitch with Vycril 1/0 (Fig. 6). Extracting the foreign bodies proved challenging through its entrance hole, which required discreet enlargement due to the reversed barb tips hindering extraction. Upon exploring the entrance wound, we found that the spears' trajectory was upward, passing through the 11th intercostal space to penetrate the liver. After a complete revision of the cavity's organs with no evidence of any other injury, we left a tube drainage in the Morrison space. The estimated blood loss was 500 ml.

The patient follow-up was favourable, with only a wound infection at the entrance point, which was treated and healed appropriately. After 2 months, he is in good health.

## Discussion

**Figure 7:**
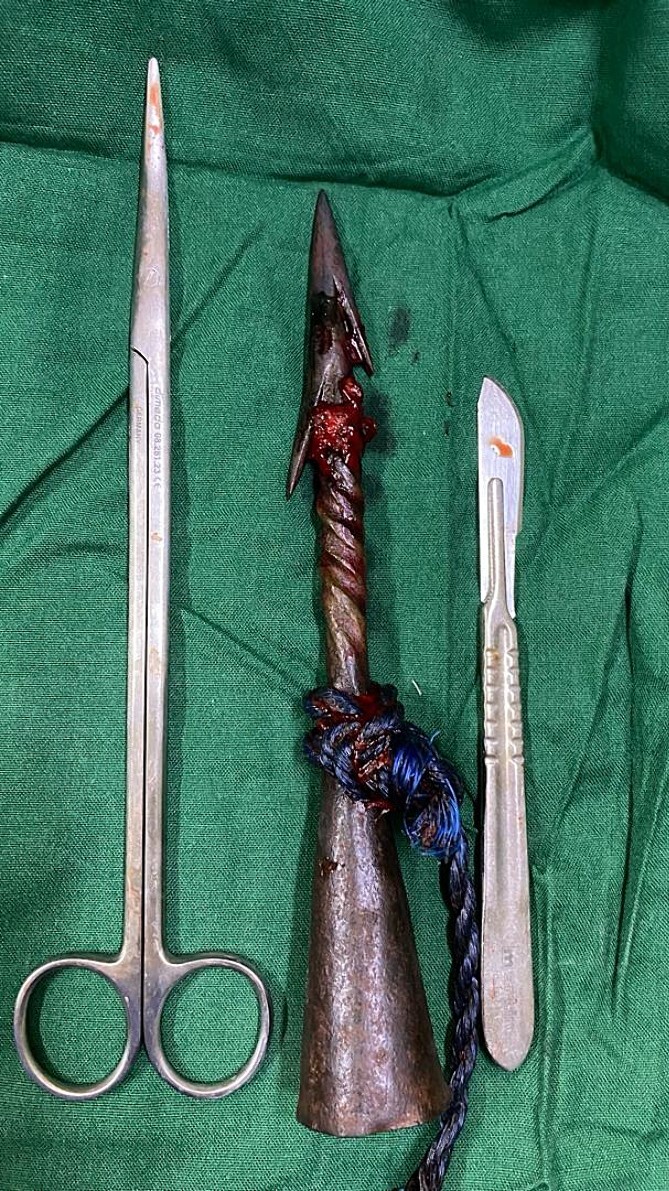
Shows the antique African iron spear after removal.

Our African elders used this antique instrument (Fig. 7) during war and rituals, and now fishers use it as a tool. During an event of violence, the fisher was stabbed by it. At the time of preparation of this report, we have not found any reports of stacking with this type of object; however, there have been occasional reports of abdominal, thoracic, and thoracoabdominal impalement using harpoons, metal bars, and other devices [1–5].

Patients with this type of injury face an immediate risk to life due to increased bleeding and subsequent hypovolemic shock; however, those who are promptly transferred to the hospital and arrive alive have a good chance of surviving [3–5]. In our instance, the patient did not show up until a day after the trauma because of transportation issues imposed by the position of the impaled object, distance, and financial constraints; fortunately for him, he remained hemodynamically stable through the time. For stable patients with penetrating trauma, contrast-enhanced CT is currently the best imaging modality with very high specificity and sensitivity for damage to the hollow and solid organs [6]. Unfortunately, our patient did not have the money to get this done, so we could only use the physical exam, X-rays, and ultrasound to make a diagnosis.

It is crucial to avoid extracting the impaled object outside of the operating room when caring for patients who have been impaled. The piercing item in situ tamponades the surrounding vascular structures, reducing bleeding [6]; this was advantageous for our patient and prevented additional damage during the extraction in the presence of the posterior barbs, which were carefully extracted through the hepatotomy.

The dimensions and trajectory of the blunt object determine the patient's positioning on the operating table, and it is a challenge for anaesthetists and surgeons [6–8]. To prevent any unintentional movement of the object and make extraction easier, we position the patient in a supine decubitus position, with the right side of the thorax protruding from the edge of the operating table.

The incision should provide a clear view of the item and surrounding anatomical tissues to ensure a safe extraction without causing further harm [7], which is why we chose the rooftop incision. Fortunately, our patient's spear tip was only a few centimetres from the liver capsule within segment V, necessitating only hepatotomy and hepatorraphy for extraction. Two cases have reported liver lateral sectionectomy and conservative care [5, 8]. It is interesting to notice that the literature evaluations do not focus much on the idea that impalement liver damage is a distinct type of injury that requires a different approach to care and that further research is necessary in this area. Managing patients with hemodynamically stable liver stacking poses several challenges but can be managed appropriately.
